# Spatial considerations during cryopreservation of a large volume sample

**DOI:** 10.1016/j.cryobiol.2016.05.013

**Published:** 2016-08

**Authors:** Peter Kilbride, Stephen Lamb, Stuart Milne, Stephanie Gibbons, Eloy Erro, James Bundy, Clare Selden, Barry Fuller, John Morris

**Affiliations:** a Institute for Liver and Digestive Health, Royal Free Hospital Campus, UCL, London, NW3 2PF, UK; b Asymptote Ltd. St. John’s Innovation Centre, Cowley Road, Cambridge, CB4 0WS, UK; c Department of Surgery, Royal Free Hospital Campus, UCL, London, NW3 2PF, UK

**Keywords:** Bioartificial liver, Large volume cryopreservation, Progressive solidification, HepG2, ELS, Encapsulated Liver Spheroids, PS, Progressive Solidification, BAL, Bioartificial Liver Device, UW, University of Wisconsin Solution (Viaspan), CPA, Cryoprotective Additive

## Abstract

There have been relatively few studies on the implications of the physical conditions experienced by cells during large volume (litres) cryopreservation – most studies have focused on the problem of cryopreservation of smaller volumes, typically up to 2 ml.

This study explores the effects of ice growth by progressive solidification, generally seen during larger scale cryopreservation, on encapsulated liver hepatocyte spheroids, and it develops a method to reliably sample different regions across the frozen cores of samples experiencing progressive solidification.

These issues are examined in the context of a Bioartificial Liver Device which requires cryopreservation of a 2 L volume in a strict cylindrical geometry for optimal clinical delivery. Progressive solidification cannot be avoided in this arrangement. In such a system optimal cryoprotectant concentrations and cooling rates are known. However, applying these parameters to a large volume is challenging due to the thermal mass and subsequent thermal lag. The specific impact of this to the cryopreservation outcome is required.

Under conditions of progressive solidification, the spatial location of Encapsulated Liver Spheroids had a strong impact on post-thaw recovery. Cells in areas first and last to solidify demonstrated significantly impaired post-thaw function, whereas areas solidifying through the majority of the process exhibited higher post-thaw outcome. It was also found that samples where the ice thawed more rapidly had greater post-thaw viability 24 h post-thaw (75.7 ± 3.9% and 62.0 ± 7.2% respectively).

These findings have implications for the cryopreservation of large volumes with a rigid shape and for the cryopreservation of a Bioartificial Liver Device.

## 1 Introduction

With the increased interest in producing bio-artificial and re-cellularized tissue scaffolds in medical settings, the cryopreservation of complex shapes becomes more significant. Large replacement and temporary support organs or biomasses are often required without delay, yet the reality is that it can take many months to produce them. Just-in-Time manufacture is not possible – neither logistically nor economically. Cryopreservation offers a solution to this dilemma as devices can be produced and stored to be thawed on demand [1], [2], [3]. This prospect makes the study of the physical implications of large volume cryopreservation and the impact of its physical parameters salient issues for research.

The primary focus of most studies on cryopreservation to date have been of relatively small volumes, typically in cryovials. These experience minimal spatial variation in their thermal cryopreservation histories & duration as no part of the sample is more than 5 mm from the surface of the vial such that heat transfer is relatively rapid. Large inhomogeneous thermal profiles tend to be minimized and disperse relatively quickly. During cooling, cryovials generally cool below their equilibrium melting point prior to ice nucleation, this results in a dendrite ice structure through the sample [4]. These parameters are not directly relevant to larger volume studies; due to thermal lag in larger volumes directional solidification occurs [4].

The present study examines cryopreservation of a 2 L biomass in a cylindrical geometry.

In the long term it is anticipated that a fluidized bed bioreactor BAL, will employ the same rigid cylindrical biomass chamber for both the cell culture and patient treatment phase, this study examined the impact of using this chamber geometry for cryopreservation.

**Fig. 1:**
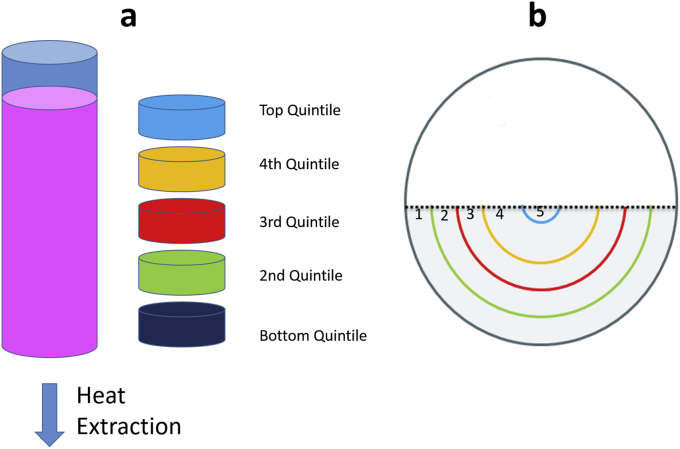
(a) A schematic of a 6 ml vial designed to produce PS (progressive solidification). Heat is extracted only from the base of the vial (shown by the arrow) with no thermal transfer through the other edges, and so the sample progressively solidified upwards. Prior to thaw, the cells were removed from the vial and dissected into quintiles as shown to the right of the vial. (b) A schematic of the face of the BAL chamber is shown to the right. As this biomass is cooled from the edges, each semi-circle line represents equal cryopreservation conditions. The maximum biomass depth was 7 cm (from the number ‘5’ in the figure directly down).

**Fig. 2:**
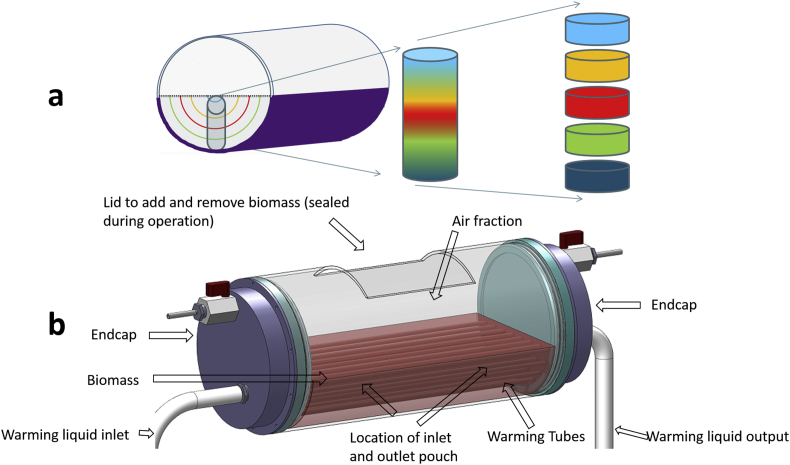
(a) A schematic of the BAL chamber with representative pouch which contained cells. These pouches were nylon mesh, permeable to culture medium and ice but impermeable to ELS. This chamber was cooled from the edges (as indicated in purple), and ice developed radially to the central semi-circle, with the semicircles representing areas that solidified at the same time. The biomass fill is represented by the dotted black line. This pouch was extracted and dissected into 5 as shown on the right of the figure. These sections were thawed consistently to determine spatial differences in damage on cooling. This approximately replicated conditions in a vial shown in Fig. 1. (b) – the large chamber used for thawing experiments. This was cooled from the edges as the BAL chamber. 25 warming tubes were passed through the biomass (indicated in red), with ethanol equally distributed through each tube using larger endcaps. The opening at the top was used for addition and removal of vials, and was sealed prior to cryopreservation. Pouches were placed within 5 cm of either the inlet or outlet tubes, with pouches of cells nearer the warming inlet thawing more rapidly. Pouches were removed for viability studies on thaw. Both BAL set-ups had a diameter of 15 cm and length of 30 cm.

The chamber used in this study has a total volume of 5 L, however the biomass component is 2 L and so residual culture medium was drained off and replaced by air to reduce thermal mass, as shown in Fig. 1, Fig. 2. In larger samples such as the one used in this study cryopreservation conditions are spatially dependent. HepG2 cells are immobilized due to their encapsulation in alginate and sedimentation of cells is not an issue in this study. Biomass near the chamber wall (within 2–3 mm) will supercool before nucleation. The remainder of the sample will not supercool, rather it will cool asymptotically to the equilibrium freezing point before solidifying when the ice front ‘grows’ through its location (the biomass itself being fixed through gravity and alginate encapsulation). After the latent heat of solidification has been liberated, the biomass in that area will cool rapidly towards the external environment temperature [4], [5]. Ice structure here tends to be planer and structured [4]. This type of ice formation is termed progressive solidification (PS).

At larger volumes, with a low surface area to volume ratio, the thawing rates will always be slow – ice is a poor conductor of heat [6]. The thawing profile has a significant impact on the recovery profile of encapsulate liver spheroids (ELS); an earlier study showed there was a significant reduction in cell viability and function at longer thawing times [7]. An experimental warming device was constructed which allowed thawing of large volumes rapidly so as to make possible the examination of spatial effects in large volume samples.

This study examined the effect of location within a sample experiencing PS on cell membrane viability and functional outcome. These experimental designs enabled the examination of the spatial significance of cooling and thawing separately. We have previously developed a method to produce PS in smaller (6 ml) vials. Producing PS in 6 ml vials gave significant cost and time savings while replicating large volume conditions [4], when determining the post-thaw outcome on ELS. Vials have been compared to samples cooled directly in a 2 L set-up where only a portion of the volume contained cells (the remainder was cell free alginate beads). Additionally a thawing device was used to determine the impact of thawing time on ELS. These three set-ups are detailed in Fig. 1, Fig. 2.

## 2 Materials and methods

### 2.1 Cell culture and encapsulation

The production of ELS has been described in detail previously [8]. HepG2 cells (human-derived hepatocyte cell-line) were grown in monolayer culture for 7 days and passaged at 80–90% confluence. Culture medium composed of alpha-MEM medium, supplemented with 50 U/ml penicillin, 50 μg/ml streptomycin (both Invitrogen plc. Carlsbad, CA, USA), and 10% human blood plasma (National Blood Transfusion Service, UK). A suspension of 3.5 × 10^6^ cells/ml in culture medium mixed 1:1 with 2% aqueous alginate solution (FMC biopolymers, Philadelphia, USA), was passed through a jetcutter system (GeniaLab, Braunschweig, Germany) resulting in spherical droplets with a diameter of 500–550 μm, which were cross linked by ejection into a buffer containing 0.204 M CaCl_2_. These (ELS) were grown in culture medium at a ratio of beads to medium of 1:58 in a fluidized bed bioreactor in a 5% CO_2_ humidified incubator at 37 °C for 11 days, with medium changed every 2–3 days.

### 2.2 Modification of the controlled rate freezer to achieve PS in small volumes during cryopreservation

As previously described [4], a controlled rate freezer (EF600-103, Asymptote, Cambridge, UK) was modified to achieve PS during cryopreservation by the addition of a module designed to take 6 ml polypropylene vials (Sigma, St Louis, MO, USA, #Z376825, 16 mm × 57 mm). This is shown in Fig. 1.

### 2.3 Modification and application of 2 L cryopreservation chamber by adding warming device

The standard large volume cryopreservation chamber consisted of a 5 L cylindrical polycarbonate chamber with simple polycarbonate end caps, into which 2 L of alginate beads (alginate without liver spheroids) were added.

For the thawing studies this was modified by adding two larger end caps with a cavity included in each end that was not in contact with the biomass. Between these end caps 25 aluminium tubes with 7 mm diameter ran through the space occupied by the 2 L alginate beads through which warmed ethanol was passed. This design is shown in Fig. 2.

The ethanol was warmed through a heat exchanger placed in a 30 °C water bath.

### 2.4 Cryopreservation protocol for 6 ml PS samples

We previously developed a scale down process which allowed the PS to form in 6 ml samples; while still large by cryopreservation standards, allowed for economical testing.

For the current studies, 5 ml aliquots of ELS were harvested and mixed 1:1 with a freezing solution (24% Me_2_SO, 76% UW v/v) precooled to 4 °C. Once equilibrated (15 min), 90% of the excess CPA supernatant was removed, giving a final volume of 5.5 ml of 12% Me_2_SO, 38% UW, and 50% ELS in culture medium, by volume, with minimal supernatant. Icestart (1% w/v, Asymptote, Cambridge, UK) was added and sank by gravity to the base of the vial, which minimised undercooling in the sample. These vials and the CRF were cooled to 4 °C before 5 vials (containing 5.5 ml each) were placed into the module on the EF600. The EF600-103 was programmed to cool at 0.3 °C/min from 4 °C to −100 °C. The samples were held in the EF600-103 at −100 °C for 1 h after the cooling cycle was complete, before being transferred to liquid nitrogen storage for 7 days.

### 2.5 Cryopreservation protocol for 2 L PS samples without warming device

As culturing 2 L of biomass was uneconomical, 2 L of alginate beads free from cells were produced instead, and equilibrated to a final concentration of 12% Me_2_SO in UW (University of Wisconsin Solution) solution. These were cooled to 4 °C and placed horizontally into a cylindrical freezing chamber with a total volume of 5 L (including 3 L air fraction).

Into this biomass pouches which were porous to liquid and ice (but not to alginate beads) were placed. ELS to a volume of 10 ml were added to these pouches. The pouch was adjusted to transcend the entire range of cooling histories in the freezing chamber.

The freezing chamber was then placed onto a specially adapted Asymptote large volume controlled rate freezer, with a curved cooling plate shaped to the chamber walls. Samples were cooled at 0.3 °C/min from 4 °C to −100 °C. Ice developed from the edges towards the centre of the chamber. Internal cooling profiles have been published elsewhere [4].

### 2.6 Thawing and dissection of 6 ml volume PS samples

Each sample was removed from liquid nitrogen storage to a sterile hood. There the vial was submerged in 37 °C water for 25s to loosen the ice core. The sample vial lid was opened and an incision made in the vial’s base. The frozen sample was pushed out of the vial through its top while still solid. A scalpel was used to dissect the sample into 5 equal circular segments from different locations as shown in Fig. 1. These were named quintiles, with the 1st quintile having frozen first and the 5th having been in the area frozen last during the cooling cycle.

Each of these segments was thawed by addition of culture medium in 330s and re-cultured in a T175 flask.

### 2.7 Thawing of 2 litre PS sample without warming device

The freezing chamber was removed from LN_2_ and its edges warmed in warm water for 60s. The end caps of the chamber were then removed and the frozen mass extracted. The pouches containing ELS were removed from the ice using a scalpel. These were then dissected into 5 components, thawed in 330s, and re-cultured. Post-thaw assays were carried out immediately on thaw as the extraction process could not be carried out under sterile conditions.

### 2.8 Thawing of 2 L sample with warming device

To thaw, the chamber was removed from liquid nitrogen and allowed to warm in air for 20 min. Ethanol warmed to 30 °C was then passed through the chamber tubing at a rate of 4 L/min. The ethanol did not come into direct contact with the biomass.

When melting was observed, the whole set-up was shaken on a plate shaker at a rate of 20 rotations/min. When the biomass was observed to be completely thawed, the ethanol was stopped and the chamber removed to a sterile hood where spatially constrained pouches were sampled.

Samples were taken linearly across the device (5 cm from each end cap) after thawing since the pouches limited movement in this direction.

### 2.9 Post-thaw functional tests post-cryopreservation

#### 2.9.1 Viability assay

A viability assay was carried out using PI/FDA staining. First 20 μl PI (propidium iodine solution, 1 mg/ml, Sigma) and 10 μl FDA (fluorescein diacetate solution 1 mg/ml, Sigma) were added to ELS and incubated at room temperature for 90 s. Next the ELS were washed once in PBS (Invitrogen) and then florescence at 617 nm (excitation) and 520 nm (emission) measured, with 1 s and 150 ms exposure for PI and FDA staining respectively. The total FDA intensity was compared to the total PI plus FDA intensity using Nikon imaging software, giving both a cell membrane integrity and metabolic viability read-out.

#### 2.9.2 Total cell counts

A known volume of ELS was removed from alginate post-cryopreservation in 16 mM EDTA (Applichem, Darmstadt, Germany) solution before the ELS were disaggregated and a nucleic count carried out using the nucleocounter system. As HepG2 cells are mononuclear this equates to cell number.

#### 2.9.3 Enzyme-linked-immuno-sorbent-assays (ELISA)

Alpha-1-fetoprotein and alpha-1-antitrypsin production were quantified by sandwich ELISA in ELS conditioned media collected 1–3 days post-thaw. This was normalized with cell counts and compared to an unfrozen control.

Mouse monoclonal antibodies (Abcam, Cambridge, UK cat # ab10071 and ab10072) were used as a capture and as an HRP linked antibody respectively, with Applichem (cat # A6935) used for a standard curve.

#### 2.9.4 MTT

A known volume of ELS was prepared and a 0.75% w/v MTT solution (tetrazolium salt, Invitrogen) was added to the ELS. After 3 h incubation the MTT was removed and the crystal product dissolved using acidified isopropanol (4 mM HCl in propan-2-ol). Total absorbance was measured at 570 nm on an Anthos III microplate reader and quantified using MANTA software.

#### 2.9.5 Glucose and lactate measurements

Culture medium samples were taken throughout the culture process, and the glucose concentration measured with an Analox GM7 device using oxidase enzyme reactions (using Analox, London, UK reagent GMRD-002A). This was then related to glucose consumption per sample.

The Analox GM7 was also used for lactate measurements on medium samples using an l-Lactate oxidase reaction using reagents GMRD 092A and GMRD 092B, from Analox. This related to total consumption per condition.

### 2.10 Statistics

To determine significance, an appropriate Student’s t-test was performed. Significance was determined at P < 0.01 unless otherwise stated. Samples for cell functional analysis contained five replicates unless indicated differently.

## 3 Results

### 3.1 Progressive solidification in 6 ml samples

#### 3.1.1 Viability and viable cell number

**Fig. 3:**
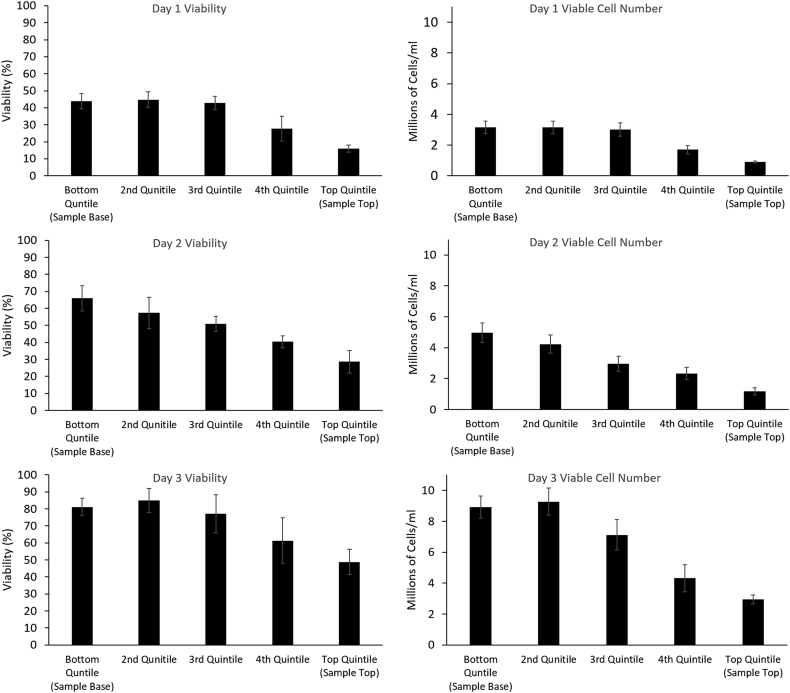
Viability (left) and viable cell number (right) of samples experiencing PS in 6 ml vials, day 1, 2, and 3 post-thaw (top, middle, and bottom respectively). All sets recover from a nadir at 1 day post thaw, By 3 days post-thaw, viability is significantly worse in the top (5th) quintile compared to viability in any of 1st (bottom) to 3rd quintiles. No significant difference in viability is observed between quintiles 1–4, 3 days post thaw. Viable cell number 3 days post thaw is significantly worse in the 5th quintile compared to all other locations. All n = 5 ± SD. Significance defined as P < 0.01 using an unpaired Student’s t-test.

Fig. 3 shows viability and viable cell number for samples in 6 ml vials experiencing PS on cryopreservation from days 1–3 post-thaw. There is a trend for highest viability and viable cell number to appear in quintiles 1–3, which then falls to a minimum in quintile 5 (top of sample).

On day 1 post-thaw, quintile 3 has a viability and viable cell number of 42.8 ± 3.9% and 3.0 ± 0.4 million cells/ml respectively compared to 15.9 ± 2.0% and 0.9 ± 0.1 million cells/ml for quintile 5. This improves to 77.1 ± 11.3% and 7.1 ± 1.0 million cells/ml for quintile 3 and 48.8 ± 7.5% and 2.9 ± 0.3 million cells/ml for quintile 5 on day 3 post-thaw. Viable cell number was significantly (P < 0.01) lower in the 5th quintile compared to all other quintiles at all time points. Viability was significantly (P < 0.01) lower in the 5th quintile compared with all other quintiles on days 2 and 3 post-thaw, and significantly lower than quintiles 1,2, and 3 on day 3 post-thaw.

### 3.2 Protein production

**Fig. 4:**
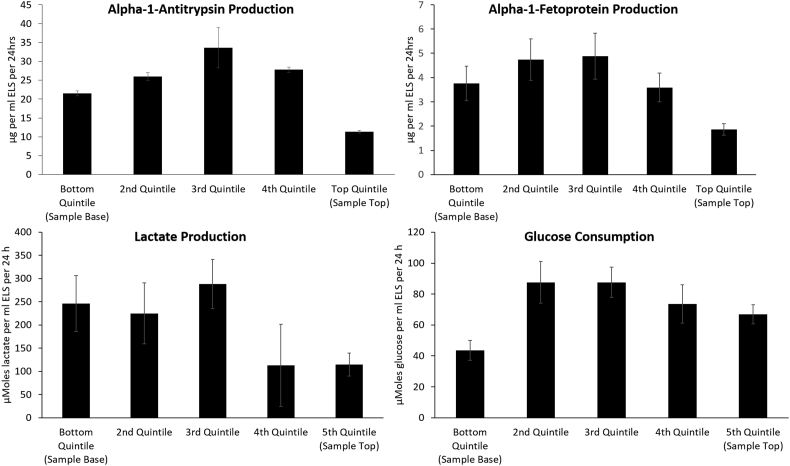
Functional outcome of ELS experiencing PS on cooling in 6 ml vials, 2 days post-thaw. Top left: alpha-1-antitrypsin production per ml ELS per 24 h. Top right: alpha-1-fetoprotein production per ml ELS per 24 h. Bottom left: lactate production per ml ELS per 24 h, and bottom right: glucose consumption per ml ELS per 24 h. In all sets the 3rd quintile has significantly increased function over the 5th quintile (P < 0.05 AFP, P < 0.01 else, unpaired Student’s T-test). Protein production average of n = 3 ± SD, repeated twice. Lactate and glucose average of n = 5 ± SD.

Fig. 4 shows the production of alpha-1-antitrypsin and alpha-1-fetoprotein for the 24 h starting one day post-thaw. These are proteins normally produced by ELS, and it is essential that the ELS protein producing functions recover post-thaw in order to make the BAL a viable treatment.

The highest productions of the tested proteins were observed at quintile 3, with the extremes of the sample (quintile 1 and quintile 5) showing lower post-thaw production. Alpha-1-antitrypsin production was 21.5 ± 0.6, 33.6 ± 5.3, and 11.4 ± 0.4 μg alpha-1-antitrypsin per ml ELS per 24 h for the 1st, 3rd and 5th quintiles respectively. Alpha-1-fetoprotein production was 3.6 ± 0.7, 4.9 ± 0.9, and 1.9 ± 0.2 μg per ml ELS per 24 h for the 1st, 3rd and 5th quintiles respectively. Alpha-1-antitrypsin production was significantly higher in the 3rd quintile than in both the 5th and 1st quintile (P < 0.01). Alpha-1-fetoprotein production was significantly higher in the 3rd quintile compared with the 5th quintile (P < 0.05).

### 3.3 Glucose consumption and lactate production

Fig. 4 shows glucose consumption and lactate production in 6 ml cryopreserved PS samples 2 days post-thaw. Glucose consumption was 246 ± 60, 288 ± 53, and 115 ± 25 μMoles of glucose per ml ELS per 24 h for the 1st, 3rd and 5th quintiles respectively. Lactate production was 44 ± 7, 88 ± 10, and 67 ± 6 μMoles of lactate per ml ELS per 24 h for the 1st, 3rd and 5th quintiles respectively.

The 1st and 5th quintiles have significantly lower (P < 0.01) lactate production than the 3rd quintile. Glucose consumption in the 5th quintile is significantly lower than in the 3rd quintile (P < 0.01).

### 3.4 Progressive solidification in a pouch in a 2 L volume

#### 3.4.1 Viability and viable cell number

Viability in samples cooled as part of a 2 L mass of alginate beads immediately post-thaw is in the 80–90% range for quintiles 1–4 of the sample. The viability drops significantly (P < 0.01) to 39.8± 18.1% at the biomass top (5th quintile) of the sample as can be seen in Fig. 4.

**Fig. 5:**
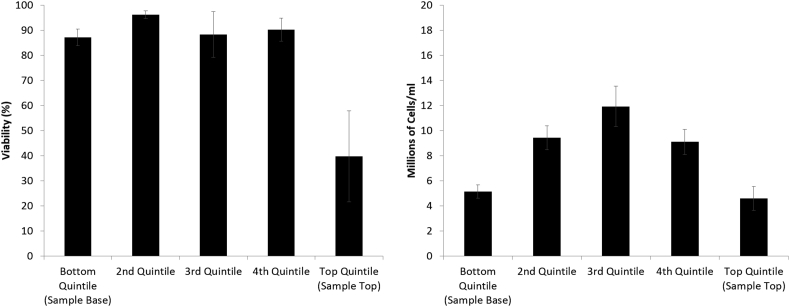
Viability (left) and viable cell number (right) of samples cryopreserved in porous pouches in a 2 L mass of alginate beads. Assessments made immediately post-thaw. Viability in the top (5th) quintile was significantly lower than in any of quintiles 1–4. No significant difference in viability was found between any of quintiles 1–4. Viable cell number is significantly lower in the 1st and 5th quintile compared with the 3rd quintile. n = 5 ± SD, significance defined as p < 0.01 using an unpaired Student’s t-test.

Fig. 5 shows that the highest viable cell density is found in the 3rd quintile, at 11.93 ± 1.61 million cells/ml ELS. This declines significantly (P < 0.01) towards the extremes of the sample, with 5.91 ± 0.54 and 4.6 ± 0.95 million cells/ml recorded in the 1st and 5th quintiles respectively.

### 3.5 MTT

**Fig. 6:**
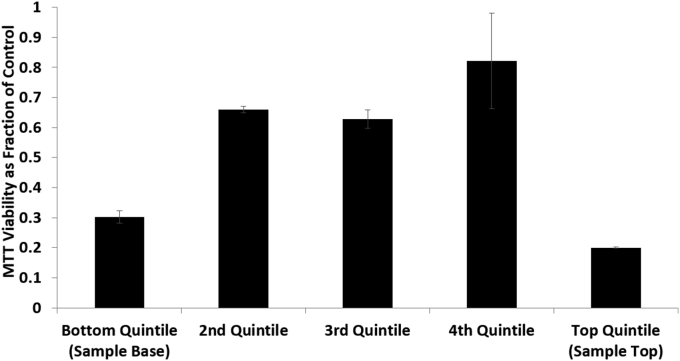
MTT viability of samples cryopreserved in porous pouches in a 2 L mass of alginate beads as a fraction of an unfrozen control. Assessments made immediately post-thaw. Quintiles 2–4 had significantly improved MTT viability compared with the top and bottom quintiles. n = 5 ± SD, significance defined as p < 0.01 using an unpaired Student’s T-test.

The pattern for MTT activity showed no significant difference in cell function between the 2nd, 3rd, and 4th quintiles, with function between 0.6 and 0.85 MTT absorbance units per ml ELS of the unfrozen control. Function fell at the edges, with 0.30 ± 0.02 and 0.20 ± 0.002 fractions of the unfrozen control measured (significantly worse than quintiles 2–4, P < 0.01); these were both significantly worse than the 2^nd^–4th quintiles, with the 1st quintile being significantly better than the 5th (P < 0.01), Fig. 6.

### 3.6 Thawing of a 2 L volume

**Fig. 7:**
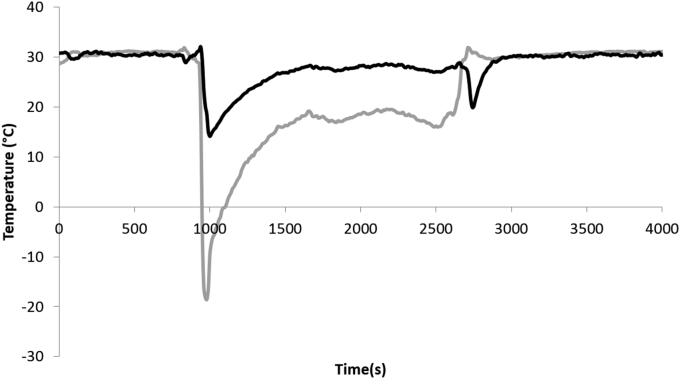
Temperatures measured in the warming fluid (ethanol) entering (black) and leaving (grey) the thawing tubes of the chamber with warming device. Ethanol was equilibrated to 30 °C as the chamber warms in air, after 900s the ethanol was pumped through the thawing tubes at 4 L/minute. The temperature difference stabilized at around 10 °C, with average temperature rising as the biomass warms. Most of the alginate bead and ELS has thawed by 2600s, where the flow was briefly reversed to dislodge remaining ice. The chamber was removed from the warming circuit after 3000s.

Fig. 7 shows thermal profiles in the inlet and outlet tubes of the warming ethanol fluid. The pre-warmed ethanol started to be pumped through the warming device after 900s. The initial temperature difference between the ethanol going into and coming out of the chamber was 33 °C, though it rapidly equilibrates to a 10 °C difference. After 1700s of ethanol flowing through the device, areas near the flow output were observed frozen while the majority of the sample had thawed. To complete thawing, the flow direction was reversed for the final 300s.

The entire thawing process took 50 min, 35 of which used ethanol warming.

### 3.7 Warming rates impact on viability and viable cell number

**Fig. 8:**
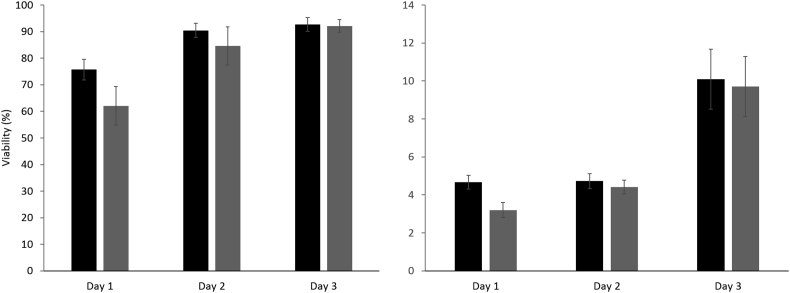
Viability (left) and viable cell number (right) of samples near the inlet (black), or outlet (grey) days 1–3 post thaw. Both viability and viable cell number are significantly better in samples nearer the warmer inlet 1 day post-thaw. No significant difference is observed in any set at any other time point. n = 5 ± SD, significance defined as p < 0.01, using an unpaired Student’s T-test.

On thaw, samples were taken from pouches 5 cm from the inlet of the flow and 5 cm from the flow outlet and re-cultured, results shown in Fig. 8. At 24 h post thaw, ELS thawed near the inlet had viability significantly (P < 0.01) higher than the outlet, at 75.7± 3.9% and 62.0± 7.2% respectively. The viable cell numbers at the entrance were also significantly (P < 0.01) higher, at 4.7 ± 0.4 million cells/ml and 3.2 ± 0.4 million cells/ml for samples near the inlet and outlet respectively.

## 4 Discussion

Our results show that the heterogeneous spatial conditions characterized during cryopreservation determine function post-thaw. In terms of viability and viable cell number, there is a general decline in outcome days 1–3 post-thaw from the 1st to 5th quintile. Functional assessments confirm this, with the exception of the first area to solidify (1st quintile) which displays larger damage than those areas solidifying later (2nd to 4th quintiles) in the process. The 5th quintile, the final area to solidify on cooling had the lowest function of all. This is consistent between samples cooled either in a 6 ml or 2 L volume, each experiencing PS, and between several different post-thaw functional assays, indicating that it is a direct consequence of the process of ice formation and not simply volume. Viable cell number is seen to increase rapidly post-thaw. This is from a combination of non-viable (membrane permeable) cells recovering, as well as from rapid post-thaw proliferation and indicates that cells are able to recover quickly from cryopreservation stresses.

The lower functional outcome in the 1st quintile of the samples was expected as biomass in this location experienced supercooling, with subsequent rapid formation of ice (localized to the edge region in a larger volume) on nucleation, and a distinct temperature discontinuity. these conditions are harmful to many cell types [1], [9] including ELS [9], [10]. Localized warming would have also occurred in this region during sample extraction from the vial, which may also have had a negative impact on post-thaw outcome. The discontinuity between simple viability and cell function has been observed before in this system [7] undergoing cryopreservation, and highlights that simple cell counts or viabilities are not sufficient to mark cryopreservation success. The data indicate that the cells in the 1st area to solidify tend to survive cryopreservation relatively well, but they have greater per-cell damage resulting in lower functional outcome. This contrasts with the 3rd quintile, which tends to have equally good post-thaw cell viability, but much greater per-cell performance indicative of lower levels of damage.

The central regions (2nd to 4th quintile) experienced favourable conditions on cryopreservation, likely due to the combination of optimal cooling rates and ice formation, but without the detrimental effects of supercooling. Studies agree that reducing supercooling is beneficial to post-thaw outcome, hence the inclusion of ice nucleators or manual nucleation in many freezing protocols [9], [10], [11]. As the biomass further away from the chamber edge will not experience supercooling (rather a slowly expanding ice front at the equilibrium freezing point) good viability in this central region agrees well with these data.

The last region (5th quintile) to solidify in the biomass has a much worse post-thaw outcome. It is well reported that the last region to solidify in cylindrical or spherical containers – the most common and efficient approximate shape for large biological samples – experiences a rapid reduction in temperature on freezing [4], [5]. This deviates from the optimum cooling conditions on cryopreservation.

Furthermore, as the cryoprotectant Me_2_SO is toxic at long exposure times and high concentrations in the liquid state [1], [12], [13], the last area to freeze will endure the largest CPA toxicity linked injury. The effects of solute-redistribution on solidification, which will cause the freezing solution to depart from its optimal freezing concentrations, must also be taken into account. This solute-redistribution will cause increasing damage with increased distance from the ice nucleation point, and is greater with the slow rate of ice growth seen in our system [14], [15]. Damage in this section could perhaps be mitigated by adding more excess supernatant above the biomass. This will reduce the rapid temperature fall after solidification, and it will also provide space that the freeze concentrated material can dilute into. A balance must be struck between excess volume and acceptable cryopreservation conditions – a larger total volume will reduce practical cooling and warming rates.

Data for the large scale freezing largely agreed with that seen in smaller 6 ml PS samples for the central regions (quintiles 2–5). Lower viable cell number is observed in quintile 1 in 6 ml samples compared with large volumes – this is likely an artifact of poor warming as the sample edges were warmed slightly to extract the biomass. The overall viability is higher on average as these data points were taken on thaw and so delayed onset cell death will not have impacted on viability [3], [10]. As samples cooled without using the thawing device were thawed equally, the heterogeneous outcome can be linked with the physical differences on cooling.

The impact of small differences in warming rates is distinctly visible from Fig. 8. At day 1 post-thaw samples 5 cm from the outlet of the warming tubes having only 2/3 of the viable cell number of those 5 cm from the inlet. This is surprising as the thawing time was not dissimilar (only 5 min difference in 50 min of thaw time), and it emphasizes the importance of consistent and rapid thawing for successful post-thaw outcome of large volume cryopreservation. The flow time of the warming fluid through the system was 1.5 s; having faster thaw time to minimize spatial variation on thaw was difficult to achieve practically. For optimal cryopreservation, this data suggests that novel, faster, and more consistent thawing methods need to be developed to realize optimal large volume cryopreservation.

Despite there being fewer studies in the literature, warming may just prove to be as important as cooling rates in developing successful cryopreservation procedures. Rapid warming is generally favored in protocols, though this can only be readily achieved in small volumes, such as cryo-straws and water baths, or with compressed cryobags [2], [12]. With the advent and need for large volume cryopreservation, this problem is starting to be addressed. Most extant work focuses on tissue grafts cooled in traditional ice forming cryopreservation where cracking or tearing can occur with non-optimal thawing protocols, or it examines warming large vitrified samples where cracking must also be avoided but rapid thawing is essential to avoid ice nucleation [13], [16], [17], [18], [19]. It has been posited that methods such as nanoparticle and dielectric warming may be required for more rapid thawing strategies [13], [17], [20].

These data highlight that spatial dimensions become very important when considering successful cryopreservation with PS present. In this study, cells have been immobilized through their encapsulation in alginate, so localized effects can be studied accurately without mixing of biological material. The same is true for complete organs, where cells rigidly adhere to the intercellular matrix.

## 5 Conclusion

This study has shown there exists a region in samples experiencing progressive solidification on freezing that optimized post-thaw function of ELS.

The last quintile in the sample to solidify displays a significantly worse outcome. While this is not ideal, the cylindrical nature of the bioartificial liver means that the last quintile to solidify (measured by radius) contains less than 4% of the total biomass and so its impact on total outcome is minimal. The 1st and 2nd quintiles contain approximately half of the biomass. Usage of the same chamber for cryopreservation as is used in the cell culture and treatment phases is possible, though challenges remain.

For large volume thawing, perfusing warm fluid through a system may not be sufficient, and new methods will have to be utilized to optimize cryopreservation strategies.

This study demonstrated that functional outcome of a biomass may vary intra-sample on the large scale, an important consideration for all large volume cryopreservation. In addition, thawing profiles can be as important as cooling profiles and must be controlled effectively for rapid recovery of large scale cryopreserved biomasses.

## Conflict of interest

No conflict of interest has been identified in this study.

## References

[1] Hengstler J.G., Utesch D., Steinberg P., Platt K., Diener B., Ringel M., Swales N., Fischer T., Biefang K., Gerl M., Bottger T., Oesch F. (2000). Cryopreserved primary hepatocytes as a constantly available in vitro model for the evaluation of human and animal drug metabolism and enzyme induction. Drug Metab. Rev..

[2] Maurel P. (2010). Hepatocytes: Methods and Protocols.

[3] Sosef M.N., Baust J.M., Sugimachi K., Fowler A., Tompkins R.G., Toner M. (2005). Cryopreservation of isolated primary rat hepatocytes – enhanced survival and long-term hepatospecific function. Ann. Surg..

[4] Kilbride P., Morris G.J., Milne S., Fuller B., Skepper J., Selden C. (2014). A scale down process for the development of large volume cryopreservation. Cryobiology.

[5] Meryman H.T. (1966). Cryobiology.

[6] Slack G.A. (1980). Thermal conductivity of ice. Phys. Rev. B.

[7] Massie I., Selden C., Hodgson H., Fuller B., Gibbons S., Morris G.J. (2014). GMP cryopreservation of large volumes of cells for regenerative medicine: active control of the freezing process. Tissue Eng. Part C. Methods.

[8] Erro E., Bundy J., Massie I., Chalmers S.A., Gautier A., Gerontas S., Hoare M., Sharratt P., Choudhury S., Lubowiecki M., Llewellyn I., Legallais C., Fuller B., Hodgson H., Selden C. (2013). Bioengineering the liver: scale-up and cool chain delivery of the liver cell biomass for clinical targeting in a bioartificial liver support system. BioRes. Open Access.

[9] Morris G.J., Acton E. (2013). Controlled ice nucleation in cryopreservation–a review. Cryobiology.

[10] Massie I., Selden C., Hodgson H., Fuller B. (2011). Cryopreservation of encapsulated liver spheroids for a bioartificial liver: reducing latent cryoinjury using an ice nucleating agent. Tissue Eng. Part C. Methods.

[11] Gunasena K.T., Villines P.M., Critser E.S., Critser J.K. (1997). Live births after autologous transplant of cryopreserved mouse ovaries. Hum. Reprod..

[12] Heidemann R., Lunse S., Tran D., Zhang C. (2010). Characterization of cell-banking parameters for the cryopreservation of mammalian cell lines in 100-ML cryobags. Biotechnol. Progr..

[13] Wusteman M., Robinson M., Pegg D. (2004). Vitrification of large tissues with dielectric warming: biological problems and some approaches to their solution. Cryobiology.

[14] Korber C. (1988). Phenomena at the advancing ice liquid interface – solutes, particles and biological cells. Q. Rev. Biophys..

[15] Miyawaki O., Liu L., Nakamura K. (1998). Effective partition constant of solute between ice and liquid phases in progressive freeze-concentration. J. Food Sci..

[16] Wang T., Zhao G., Liang X.M., Xu Y., Li Y., Tang H., Jiang R., Gao D. (2014). Numerical simulation of the effect of superparamagnetic nanoparticles on microwave rewarming of cryopreserved tissues. Cryobiology.

[17] Ruggera P.S., Fahy G.M. (1990). Rapid and uniform electromagnetic heating of aqueous cryoprotectant solutions from cryogenic temperatures. Cryobiology.

[18] Jashari R., Van Hoeck B., Ngakam R., Goffin Y., Fan Y. (2013). Banking of cryopreserved arterial allografts in Europe: 20 years of operation in the European homograft bank (EHB) in brussels. Cell Tissue Bank..

[19] Wassenaar C., Wijsmuller E.G., Van Herwerden L.A., Aghai Z., Van Tricht C.L., Bos E. (1995). Cracks in cryopreserved aortic allografts and rapid thawing. Ann. Thorac. Surg..

[20] Evans S. (2000). Electromagnetic rewarming: the effect of CPA concentration and radio source frequency on uniformity and efficiency of heating. Cryobiology.

